# Effects of Brain Breaks Videos on the Motives for the Physical Activity of Malaysians with Type-2 Diabetes Mellitus

**DOI:** 10.3390/ijerph17072507

**Published:** 2020-04-06

**Authors:** Aizuddin Hidrus, Yee Cheng Kueh, Bachok Norsaádah, Yu-Kai Chang, Tsung-Min Hung, Nyi Nyi Naing, Garry Kuan

**Affiliations:** 1 Unit of Biostatistics and Research Methodology, School of Medical Sciences, Universiti Sains Malaysia, 16150 Kubang Kerian, Kelantan, Malaysia; aizuddinh88@gmail.com (A.H.); norsaadah@usm.my (B.N.); 2 Community and Family Medicine Department, Faculty of Medicine and Health Science, Universiti Malaysia Sabah, 88400 Kota Kinabalu, Malaysia; 3 Department of Physical Education, National Taiwan Normal University, Taipei 24449, Taiwan; yukaichangnew@gmail.com (Y.-K.C.); ernesthungkimo@yahoo.com.tw (T.-M.H.); 4 Institute of Research Excellence in Learning Science, National Taiwan Normal University, Taipei 24449, Taiwan; 5 Faculty of Medicine, Universiti Sultan Zainal Abidin, Medical Campus, Jalan Sultan Mahmud, 20400 Kuala Terengganu, Terengganu, Malaysia; syedhatim@unisza.edu.my; 6 Exercise and Sports Science Programme, School of Health Sciences, Universiti Sains Malaysia, 16150 Kubang Kerian, Kelantan, Malaysia; 7 Department of Life Sciences, Brunel University, London UB8 3PH, UK

**Keywords:** Brain Breaks^®^, video exercise, IPAQ, PALMS, motivation, sport, repeated measure

## Abstract

Brain Breaks videos are web-based structured physical activity (PA) videos that aim at stimulating an interest in learning and promoting health. Exercise is one of the important treatment regimens for people with type 2 diabetes mellitus (T2DM). Thus, the objective of this study was to determine the effects that Brain Breaks videos have on the motives for PA, as measured by the Physical Activity and Leisure Motivation Scale-Malay (PALMS-M), and the amount of PA, as measured by the International Physical Activity Questionnaire-Malay (IPAQ-M), in T2DM patients (the most common type of diabetes mellitus patients). This study was conducted using a randomized, double-blind design and grouped subjects under two research conditions: an experimental group given Brain Breaks videos and a control group. Purposive sampling was employed to recruit 70 T2DM patients (male = 39, female = 31) with the mean age of 57.6 (SD = 8.5) from Hospital Universiti Sains Malaysia, Kelantan. Over a four-month period, the participants in the experimental group were asked to perform PA daily based on a Brain Breaks video (10 min in duration) that was shared through a WhatsApp group. All participants from both groups answered the PALMS-M questionnaire five times: pre-intervention, the end of the first month, second month, and third month, and post-intervention. A repeated measure multivariate analysis of variance and a repeated measure analysis of variance were performed for the analyses of the data. The results demonstrated that four (appearance, others’ expectations, physical condition, and mastery) out of eight motives for PA produced a significant mean score difference between the two study groups. All eight motives for PA showed an upward trend for the experimental group during the study period, while the control group showed a downward trend for all motives during the study period. As for the amount of PA, both groups showed significant differences (*p* = 0.001). The amount of PA increased in the experimental group during the study period, while it decreased in the control group. Therefore, Brain Breaks videos can be considered as an effective intervention for motivating T2DM patients for PA and improving their amount of PA.

## 1. Introduction

Physical activity (PA) has been considered a cornerstone for the development and maintenance of a healthy lifestyle for centuries. In the fifth century BCE, the physician Hippocrates stated, “all parts of the body, if used in moderation and exercised in labors to which each is accustomed, become thereby healthy and well developed and age slowly; but if they are unused and left idle, they become liable to disease, defective in growth and age quickly” [1]. In the early 1970s and 1980s, the study of PA levels among populations began, and this continues to today [2]. In addition, physical inactivity was found to be one of the vital risk factors for coronary heart disease [3], and since then, concerns regarding physical inactivity have emerged as a leading health issue. Thus, doctors and researchers have initiated many studies and gathered much research related to PA and disease reduction [4,5,6]. 

Nevertheless, over 50% of Malaysians have chosen to be physically inactive rather than to improve their health levels by engaging in regular exercise [7]. Based on the National Health and Morbidity Study (NHMS) 2015, 66.5% of Malaysian adults are physically active [8]; however, among these, only 25.4% are highly active and the remainder are only minimally active. As recently reported in the NHMS 2017, only 45% of Malaysian adolescents are physically active [9]. Another study demonstrated that a group of adults who were inactive during their adolescence remained inactive into adulthood [10]. Therefore, the lack of PA among Malaysian adolescents may be indicating that Malaysians will be less physically active in the future, leading to a rise in morbidity and morbidity prevalence.

The World Health Organization (WHO) has defined diabetes as “a chronic disease that occurs either when the pancreas does not produce enough insulin or when the body cannot effectively use the insulin it produces” [11]. Type 2 diabetes mellitus (T2DM) is the most common type of diabetes worldwide, with being physically inactive and overweight as the main contributing factors [11]. The WHO has also predicted that diabetes incidence and prevalence will reach 366 million individuals by 2030. In Malaysia in the 1960s, diabetes prevalence was just 0.65% [12]. Each year, the prevalence has risen. By the early 1980s, the prevalence of diabetes had increased to 2.4%, escalating further to 8%–12% and 14% by the early 1990s and 1998, respectively [12]. In 2015, the NHMS 2015 reported that 17.5% of the >18-year-old Malaysian population (3.5 million in total) had been diagnosed with diabetes [8]. According to Tee and Yap [13], obesity and being overweight are the major risk factors for T2DM in Malaysia. These two risk factors are commonly known as being highly related to bad dietary habits and physical inactivity [14], which may be the main factors contributing to the consistent increase in diabetes, especially in T2DM, in Malaysia over the past several decades. 

Motivation has frequently been reported as one of the crucial components behind participation in sporting activities [15]. The same is true for exercise and PA, where motivation plays a major role not only in promoting involvement in PA but also in maintaining this involvement [16,17]. Vallerand [18] divided motivation into intrinsic motivation (doing something for its own sake) and extrinsic motivation (doing something as a means to achieve rewards and not for its own sake). From other perspectives, theories such as self-determination theory [19] and achievement goal theory [20] have adopted as parameters the measure of an individual’s motivation for regular PA. Intrinsic motivation and the self-determination theory of motivation both present a similar concept. Vallerand [18] stated that intrinsic motivation “refers to engaging in an activity for the pleasure and satisfaction that one experiences while learning, exploring, or trying to understand something new”, whereas the self-determination theory of motivation presents enjoyment, challenge, skill development, and mastery [21,22] as the factors that can motivate a person to be consistently physically active. As for extrinsic motivation and the achievement goal theory of motivation, both are related to goals such as receiving rewards, a healthy body, a good appearance, or any other factor that is not related to the PA itself [18,22]. Thus, to determine an individual’s desire and ability to be consistently physically active, it is important to know and understand the factors that motivate the individual to be involved in PA. 

Developed by Rogers and Morris [23], the Physical Activity and Leisure Motivation Scale (PALMS) is an instrument used to measure an individual’s motivation for PA. Previously, it consisted of 73 items and was called the Recreational Exercise Motivation Measure (REMM), but the REMM was shortened into 40 items to become the PALMS [23]. These 40 items are classified into eight different factors or motives: enjoyment, mastery, competition/ego, appearance, affiliation, others’ expectations, psychological condition, and physical condition, which appear in the version specifically for adults. There is also a version for children, in which the 40-item PALMS is shortened to 28 items with seven factors (PALMS-Y), removing others’ expectations as a factor [24]. The adult version of the PALMS was translated into the Malay language and went through confirmatory factor analysis (CFA) for validation purposes, which led to the development of the PALMS-M [25]. The CFA result for the PALMS-M indicated sound validity and reliability [25].

A study comparing the PA levels among T2DM patients showed that patients with a fasting blood sugar of less than 9mmol/L are more active during leisure and daily activities than patients with levels higher than 11mmol/L [26]. PA has been recognized as one of the proximal determinants for T2DM [27]. Hence, improving the motivation of T2DM patients for being more physically active is crucial. Presently, PA interventions are commonly performed among patients with non-communicable diseases [28,29,30,31].

Recently, one promising intervention has been developed by HopSports [32], a video exercise known as Brain Breaks^®^ Physical Activity Solutions or Brain Breaks for short. These exercises are web-based structured PA breaks that are intended to stimulate an individual’s health and learning. The exercises have been specifically designed for use in individual or group settings to motivate users to enhance their mental skills while providing the opportunity to be not only physically active but also to learn new motor skills, languages, works of art, music, and about different cultures [33]. On the Global Community Health website, educators worldwide contribute by uploading exercise videos that suit their respective customs and cultures. These videos are then shared online and are accessible to anyone who would like to implement them. Other types of Brain Breaks are simple transitional physical and mental exercises designed to equip facilitators with tools that can manage the physiology and attention of a class and keep children in the most receptive state for learning [34]. In this study, we used Brain Breaks as an intervention because it is motivational, easy, and promotes exercise and being physically active using a new innovative yet fun environment for T2DM patients. Thus, we aimed to determine the effects that Brain Breaks video exercise, with a four-month intervention period, have on the motives for PA and amount of PA in T2DM patients. 

## 2. Materials and Methods 

### 2.1. Study Design and Recruitment

The researchers decided to employ a purposive sampling as the study targeted T2DM patients. The inclusion criteria for the present study were: the T2DM patients must be 18 years old and above, understand the purpose of the present study, participate voluntarily, and be a Malaysian who is able to read and write in the Malay language and answer the questionnaires. The T2DM patients with disabilities/comorbidities that hindered them from performing PA were excluded. 

**Figure 1:**
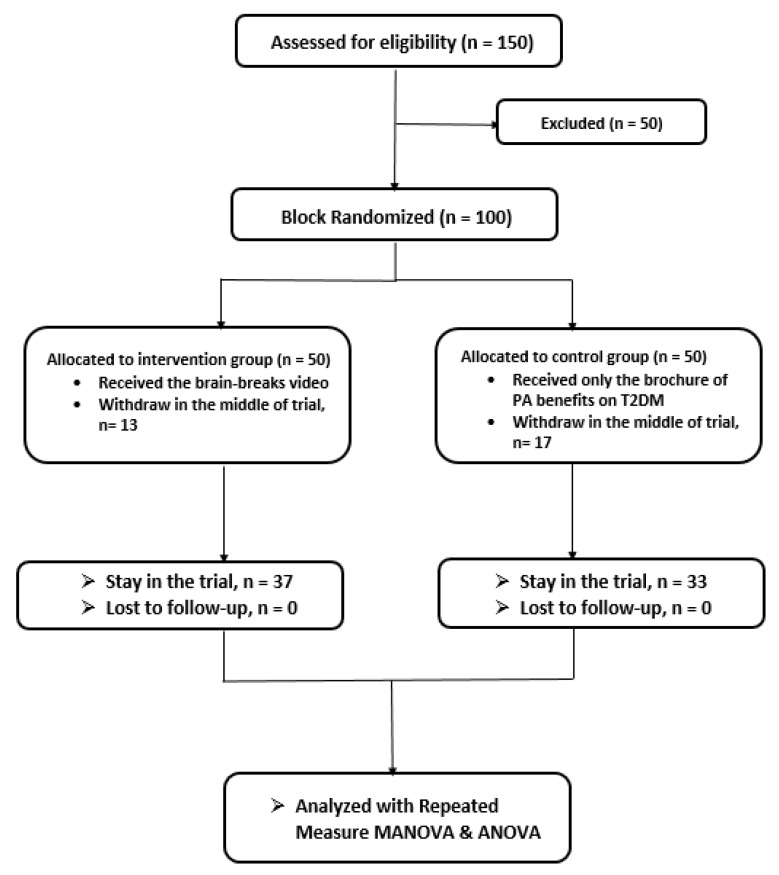
Participants group allocation diagram.

A randomized controlled trial was performed in the present study. The T2DM patients who fulfilled the inclusion criteria were then randomized into experimental and control groups using computer-generated block randomization [35]. Participants who opt out in the middle of the intervention phase were excluded in the study and the data were not included in the analysis. Flow chart of participant group allocation is illustrated in Figure 1.

### 2.2. Participants

The present study involved a total of 70 T2DM patients. There were 37 participants in the experimental group, while the control group consisted of 33 participants. The demographic data of the T2DM patients are described in Table 1, where patient age is presented as mean (standard deviation [SD]), and group, gender, and ethnicity are presented as frequency (%).

### 2.3. Procedures

This study obtained approval from the Universiti Sains Malaysia (USM) Human Research Ethics Committee (USM/JEPeM/18040201) and was conducted in accordance with the guidelines of the International Declaration of Helsinki. Participants were informed that their participation was voluntary and that they could withdraw at any time without incurring any loss or penalty. Written informed consent was obtained from each participant prior to their participation in the study.

Data collection was conducted at the Hospital USM, Kubang Kerian, Kelantan. The data collection period was from October 2018 to May 2019. Eligible participants were approached during their visit to their physician for their regular medical check-up appointment.

At baseline, both the experimental and control groups were required to answer the PALMS-M to evaluate their initial motives for PA. For the experimental group, participants were invited to join a WhatsApp group where the Brain Breaks videos were shared throughout the intervention phase. During the intervention, an exercise video of 10 min in length specifically designed for diabetes patients was uploaded to the WhatsApp group, and the participants were required to perform the exercise either outdoors or indoors (depending on their preference). They were reminded regularly as we uploaded a video every week. A different exercise video was given on the first day of each week in order to help participants avoid getting bored with the same exercise. Each of the experimental participants was given an adherence log-book for monitoring their progress. For the control group, the participants were given a brochure containing a brief introduction to the benefits of PA on health. The duration of the intervention was four months. At the end of each month, participants in both the experimental and control groups were required to answer the PALMS-M. The outcome of the study was based on the eight motives for PA as measured by the PALMS-M. 

Other than motives, we also measured the patients’ amount of PA using the IPAQ-M. Similar to the PALMS-M, the patients were also required to answer the IPAQ-M five times (pre-, end of the first month, second month, and third month, and post-) during the intervention period. 

### 2.4. Instruments

*(a)*  *Physical Activity and Leisure Motivation Scale-Malay (PALMS-M)*

The PALMS was designed to measure the motives for participating in PA or leisure activity (23). The main language spoken among the study population was Malay; therefore, the PALMS-M was used in the present study. Like the original PALMS, the PALMS-M also consists of 40 items measuring eight domains for motives for participating in PA: mastery, physical condition, psychological condition, affiliation, appearance, enjoyment, competition/ego, and others’ expectations. Each domain consists of five items scored on a 5-point Likert scale ranging from 1 (strongly disagree) to 5 (strongly agree). The PALMS-M has shown good validity with acceptable fit indices in CFA (RMSEA = 0.041 [90%CI: 0.038, 0.044], CIfitRMSEA = 1.000, CFI = 0.911, TLI = 0.091, SRMR = 0.052) [25]. The composite reliability of the PALMS-M was satisfactory, ranging from 0.65 to 0.85 [25]. Kueh et al. [25] identified two problematic items in the PALMS-M when conducting a validation study among university undergraduate students. They suggested that for future research on other study populations, the PALMS-M with 40 items should be utilized. Therefore, for the present study, the PALMS-M with 40 items and eight domains was used to measure the levels of the motives for participating in PA among people with T2DM.

*(b)*  *Malay Version of International Physical Activity Questionnaire (IPAQ-M)*

The International Physical Activity Questionnaire (IPAQ) was initially developed by the International Consensus Group consisting of four versions for the long IPAQ and four versions for the short IPAQ [36]. The short version includes seven questions, while the long version consists of 27 questions. The questions in the short version are related to the time that a person has spent being physically active in the past seven days based on four types of activity (vigorous, moderate, walking, and sitting). The IPAQ score analysis is based on the following metabolic equivalent of task (MET) values: walking = 3.3 METs, moderate activity = 4.0 METs, and vigorous activity = 8.0 METs. Recently, a reliability and validity check of the self-administered IPAQ-M was done by Chu and Moy [37]. For the short version, their results showed that the interclass correlation coefficient (ICC) ranged from 0.54 to 0.86, while the Spearman correlation coefficient ranged between 0.67 and 0.98. Based on these results, Chu and Moy concluded that the self-administered IPAQ-M is reliable and valid for adoption among Malaysian adults. Thus, the present study decided to use the short version of IPAQ-M for measuring the amount of PA among people with T2DM. 

### 2.5. Data Analysis

The data analyses were conducted using the Statistical Package for the Social Sciences (SPSS) version 25.0. The data consisted of the two groups (i.e., experimental and control) with a five-time measurement of the outcome variables (i.e., the eight domains of the PALMS-M). Repeated measure multivariate analysis of variance (RM MANOVA) was conducted to examine the effects of the Brain Breaks video exercises on the motives for PA. Repeated measure analysis of variance (RM ANOVA) was used to examine the effect of the Brain Breaks video exercises on the amount of PA. The effects examined consisted of time, group, and interaction (time x group) effects. A *p*-value of < 0.05 was taken as a significant result.

## 3. Results

### 3.1. Effects on Patients’ Motives for PA

Table 2 presents the mean scores (SD) of the participants for the eight motives in the PALMS-M for both the experimental and control groups from pre- to post-intervention. 

Table 3 shows the overall mean difference for all eight motives in the PALMS-M between the experimental and control groups. Overall, the experimental group had a higher mean for the motives than the control group. Based on the results, four out of the eight motives in the PALMS-M were significantly different between the two groups. Appearance, others’ expectations, physical condition, and mastery were the motives that showed a significant difference between the groups with *p*-values of 0.014, < 0.001, 0.023, and 0.021, respectively. However, the remaining motives of competition, affiliation, psychological condition, and enjoyment showed no significant differences between the experimental and control groups. 

From a different perspective, we compared the changes in the patients’ motives for PA from the pre- to post-intervention period for both groups. Table 4 and Table 5 show the comparison details within the experimental and control groups. Based on Table 4, six of the eight motives were significantly different from the pre- to post-intervention period for the experimental group. The competition, others’ expectations, affiliation, psychological condition, mastery, and enjoyment motives produced *p*-values of < 0.001, < 0.001, 0.047, 0.035, 0.025, and 0.009, respectively. In general, the motives increased from pre- to post-intervention period. As for the control group, Table 5 shows five motives with significant changes (competition, others’ expectations, physical condition, psychological condition, and enjoyment motives had *p*-values of 0.001, < 0.001, 0.001, 0.035, and 0.026, respectively) from the pre- to post-intervention period. In general, the motives of PA decreased from the pre- to post-intervention period.

Another comparison was performed between the two groups with regard to time (pre- to post-intervention period) as we wanted to determine the time–group interaction. Table 6 shows the details of the time–group interaction for all of the PALMS-M motives. Based on Table 6, if the mean value of one group is outside the 95% confidence interval (CI) of another group, this indicates that both groups are significantly different and vice versa. 

### 3.2. Effects on the Patients’ Amount of PA

From Table 7, it can be seen that there is a significant difference between the experimental and control groups. The experimental group produced a higher mean score in the IPAQ-M as compared to the control group, giving a *p*-value of 0.001. 

**Figure 2:**
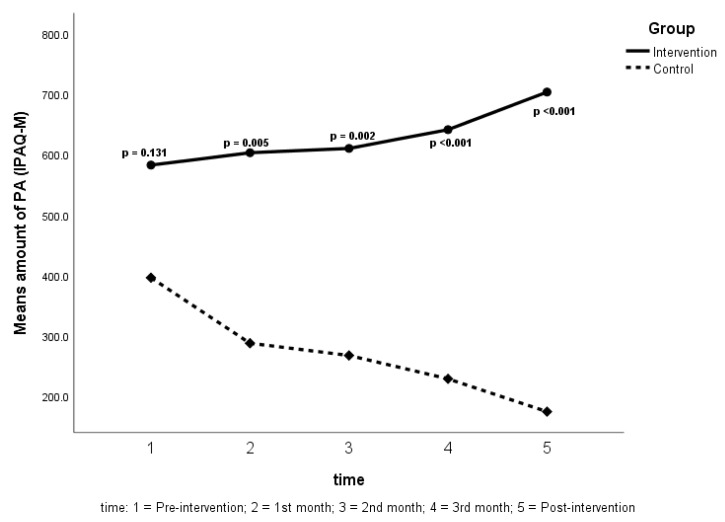
PA amount changes of both groups from the pre- to post-intervention period.

As for the time effect, the experimental group showed a significant increase in the amount of PA, while the control group showed a significant decrease. Based on Figure 2, we can see that the difference between the two groups became more significant as time went along during the experimental period. The changes in the *p*-value in comparing the two groups, from pre- to post- experimental was from 0.131 to < 0.001.

## 4. Discussion

The aim of the present study was to determine the changes in the motives of T2DM patients for PA after being given Brain Breaks videos (experimental group) to view and then to compare these changes with the changes in patients who had received no Brain Breaks videos (control group). As mentioned earlier, there were eight motives (competition, appearance, others’ expectations, affiliation, physical condition, psychological condition, mastery, and enjoyment) that were measured by the eight motives in the PALMS-M, and four of these motives (appearance, others’ expectations, physical condition, and mastery) were significantly different (the experimental group scored more highly than the control group) between the two groups after the completion of the trial period. Although only four out of the eight motives were significantly different between the two groups, the experimental group scored more highly than the control group on the remaining four motives. These results indicated that the Brain Breaks video intervention produced a positive impact on T2DM patients’ motives for PA. Moreover, based on the mean score trend of the PALMS-M motives, the experimental group showed an increasing trend, while the control group showed a decreasing trend throughout the trial period. The different graph directions for the two groups may support the statement that Brain Breaks videos can help T2DM patients improve or maintain their motives for regular PA. Adult patients with high motivation to perform regular daily PA could help them to enhance insulin action and aim for optimal glycemic and health outcomes [36].

The results of the present study differ from those of a study done by Hajar et al. [37] that applied a Brain Breaks video intervention among primary school students. Using a Malay version of the PALMS-Y to measure the children’s motives for PA, their results showed that only four motives (mastery, competition, affiliation, and physical condition) showed an increasing trend in the experimental group by the end of the trial period. As for the mean difference between the two groups, only enjoyment was significantly different (*p*-value = 0.032) between the two research conditions. We believe that the distinction between the current study and their study is most probably due to the different age groups of the participants. As reported by Biddle and Mutrie [38], the motives for PA among children and youth are focused on fun, skill development, affiliation, success, and challenge, while, for adults, the motives for PA continue to change in time. As time passes, older adults become more attracted to and motivated by PA by health benefits, relaxation, and enjoyment, while younger adults are motivated by challenge, skill development, and fitness [38]. In addition, another point of view was given by Ebben and Brudzynski [39], who stated that young adults are most commonly more motivated for PA for reasons of general health and maintaining fitness levels. Therefore, the effects of a Brain Breaks video intervention among children/youth can be as positive as those among adult patients. 

Furthermore, the motives for PA differ between active people and inactive people. A study conducted by Aaltonen et al. [40] that examined the motives for PA among active and inactive people (in their 30s) in Europe demonstrated a significant difference in the motives between these two groups. Active people were more likely than inactive people to consider mastery, physical fitness, social aspect of PA, psychological state, enjoyment, the desire to be fitter/look better than others, and appearance as crucial factors or motives that make PA more essential [40]. Only others’ expectations appeared to be more important among inactive people [40]. In contrast with the present study, while not all of the motives were significantly different between the two groups, the experimental group (active people) produced a higher mean score for all motives, including others’ expectations. Health status and cultural disparity could be the reason for the experimental group in the present study producing a higher mean score for others’ expectations. As is commonly known, the health status of T2DM patients is regularly monitored by doctors and even family members. Hence, it is acceptable for T2DM patients to perform PA as being ordered or prescribed by their doctors, which indirectly will be helping them to improve their health level. 

According to previous studies, cultural disparities are another factor that can contribute to the globally different motivations for PA [41,42,43]. In the West, an attractive appearance along with improving one’s appearance are considered as most influential motivates for PA [44]. The United Kingdom is an example of a place where “to physically feel in good shape”, “to improve or maintain health”, and “to feel a sense of achievement” are considered as the most crucial motivational factors for performing PA [38]. As for Turkish people, they place health status as the highest priority for performing PA, with enjoyment and competition motives following behind [45]. Thus, clarifying the PALMS questionnaire must be initiated in order to take into consideration cultural disparities.

Although the present study conveyed a positive result, there are limitations and weaknesses that must be highlighted. Participants were recruited only from a single hospital in Malaysia due to the time constraints faced by the researchers. The involvement of more participants from a number of hospitals in Malaysia could have helped the present study produce better results. Therefore, it is highly recommended that, for future studies, a multicenter community trial should be used to obtain a greater variety of responses from different hospitals. In addition, other than with a logbook, the researchers could not closely monitor the participants’ commitment to the given intervention. Participants may have lied or given incorrect information regarding their participation in given intervention. This situation was inevitable as most of the T2DM patients were difficult to assemble in a place where they could be observed performing the given intervention each day due to logistic and transportation restrictions. Yet, to ensure that the participants adhered to the given intervention, we sent regular reminders in daily intervals to the WhatsApp group. 

Another limitation was that the participants were restricted to only those T2DM patients who had mobile phones with internet service. This was required so the participants could install the WhatsApp application where the Brain Breaks videos were shared. With this issue and time constraints, we were unable to recruit more participants. Yet, after taking into consideration the logistic and transportation restrictions, the researchers believe that this was the most convenient way to get participants involved in the trial. Lastly, the researchers admit that only focusing on a single non-communicable disease (T2DM) could be another weakness of the present study. Hence, we urge that future studies include more types of non-communicable diseases to present more comprehensive results. 

As for the amount of PA, the researchers had difficulty in finding the best studies for comparison to the present study. This is most likely due to the relative newness of Brain Breaks videos to the global population, especially in regard to T2DM patients and the Malaysian community. Therefore, the results from the present study on the effects of the Brain Breaks videos on the amount of PA among the participants could not be discussed in detail. However, the highly significant decrease in the amount of PA in the control group can be explained by the NHMS 2015 [8], where it was reported that PA prevalence dropped from 72.3% for the 50–54 age group to 63.3% for the 55–59 age group. PA prevalence then decreased until the age group of 75+, which was only at 30%. On the other hand, the experimental group demonstrated a significant increase in the amount of PA, though not as great as the decrease in the amount of PA among the control group. Thus, a proper monitoring system for elder adults (55+ age group) on their amount of PA must be planned in order to improve their PA prevalence, especially for those with non-communicable diseases.

However, we believe that a community trial using a block randomization was an effective study design and makes the present study strong enough to convey good results. Block randomization was used to reduce selection bias, making the present study more reliable for future reference. Moreover, the researchers made three repeated measurements during the trial period (for monitoring purposes) and did regular monitoring by having conversations with the participants through WhatsApp in order to ensure that they had adhered to the given intervention. Finally, the present study was performed using a RM MANOVA for the patients’ motives for PA assessment rather than multiple RM ANOVA, which was done because MANOVA has greater power and improves the chances of detecting differences between groups than ANOVA [46].

## 5. Conclusions

Based on the results of the current study, the researchers conclude that Brain Breaks videos can be considered as an effective intervention for motivating T2DM patients to carry out PA. Appearance, others’ expectations, physical condition, and mastery motives are all important for T2DM patients, who must always consider the disease’s complications (e.g., blindness and amputations) and demonstrate improvements in blood sugar levels to their doctors. The intervention with the Brain Breaks videos also helped to demonstrate differences in the trends for mean scores between the two groups, with the experimental group showing an upward trend and the control group showing a downward trend throughout the trial period. As for the PALMS-M, it is an effective scale for measuring the motives for PA of the participants. The Brain Breaks videos were also found to be effective for improving the patients’ amount of PA, with both groups showing a significant difference after the intervention had finished. The researchers have previously suggested for future studies the involvement of more hospitals with improved sampling methods in order to include more participants. In addition, the consideration of more types of non-communicable diseases in the trial would make the results more comprehensive and more generalizable to not only a single patient population but to other patient populations as well.

## Figures and Tables

**Table 1:** Summary of the demographic information of type 2 diabetes mellitus (T2DM) patients.

Variables	Mean (*SD*)	Frequency (%)
Age	57.6 (8.5)	
Group		
Experimental		37 (52.9)
Control		33 (47.1)
Gender		
Male		39 (55.7)
Female		31 (44.3)
Ethnicity		
Malay		63 (9.0)
Chinese		4 (5.7)
Indian		1 (1.4)
Others		2 (2.9)

**Table 2:** Summary of the mean score (SD) for each motive of Physical Activity and Leisure Motivation Scale-Malay (PALMS-M).

PALMS-M Motives	Mean (*SD*)									
	Pre-Intervention		1st Month		2nd Month		3rd Month		Post-Intervention	
	Exp ^a^	Con ^b^	Exp ^a^	Con ^b^	Exp ^a^	Con ^b^	Exp ^a^	Con ^b^	Exp ^a^	Con ^b^
Competition	15.05 (0.76)	17.67 (0.56)	16.78 (0.60)	17.48 (0.55)	18.62 (0.44)	17.42 (0.54)	20.57 (0.32)	17.09 (0.52)	20.92 (0.32)	16.45 (0.46)
Appearance	17.22 (0.78)	15.21 (0.77)	17.30 (0.77)	15.12 (0.75)	17.30 (0.77)	15.06 (0.73)	17.62 (0.68)	14.94 (0.71)	18.16 (0.57)	14.58 (0.63)
Others’ expectation	14.49 (0.53)	16.36 (0.34)	16.16 (0.43)	16.18 (0.37)	18.16 (0.34)	15.88 (0.36)	20.57 (0.30)	15.55 (0.35)	20.89 (0.30)	15.09 (0.32)
Affiliation	15.43 (0.71)	15.09 (0.60)	15.51 (0.70)	15.00 (0.58)	15.51 (0.70)	15.00 (0.58)	16.11 (0.61)	14.91 (0.54)	16.68 (0.58)	14.73 (0.49)
Physical condition	19.89 (0.51)	18.67 (0.71)	19.89 (0.51)	18.42 (0.67)	19.89 (0.51)	18.27 (0.65)	20.00 (0.49)	18.03 (0.62)	20.43 (0.47)	17.52 (0.54)
Psychological condition	18.05 (0.51)	17.48 (0.72)	18.14 (0.50)	17.27 (0.68)	18.14 (0.50)	17.24 (0.67)	18.24 (0.49)	17.03 (0.62)	19.00 (0.44)	16.45 (0.53)
Mastery	16.78 (0.65)	15.00 (0.72)	16.81 (0.65)	14.94 (0.71)	16.81 (0.65)	14.94 (0.71)	17.03 (0.60)	14.88 (0.68)	17.70 (0.55)	14.58 (0.60)
Enjoyment	17.14 (0.61)	16.64 (0.70)	17.19 (0.60)	16.58 (0.69)	17.19 (0.64)	16.58 (0.69)	17.27 (0.59)	16.33 (0.62)	17.89 (0.55)	15.91 (0.56)

^a^ Experimental group, ^b^ Control group.

**Table 3:** Overall mean difference of PALMS-M motives scores between experimental and control group.

Comparison on PALMS-M Motives (Experimental and Control Groups)	Mean Difference (95% CI)	*p*-Value
Competition	1.17 (−0.19, 2.52)	0.090
Appearance	2.54 * (0.53, 4.55)	0.014
Others’ expectation	2.24 * (1.27, 3.21)	<0.001
Affiliation	0.90 (−0.81, 2.62)	0.297
Physical condition	1.84 * (0.26, 3.42)	0.023
Psychological condition	1.21 (−0.35, 2.78)	0.125
Mastery	2.16 * (0.33, 3.98)	0.021
Enjoyment	0.93 (−0.80, 2.66)	0.288

* Significant *p*-value, Repeated Measure MANOVA between two groups was performed and no post-hoc comparisons between groups was done since there were only two group involved.

**Table 4:** Comparisons of mean score of PALMS-M motives within experimental group based on time (time effect).

Comparison	Competition		Appearance		Others’ Expectation		Affiliation		Physical Condition		Psychological Condition		Mastery		Enjoyment	
	MD (95% CI)	*p*-value	MD (95% CI)	*p*-value	MD (95% CI)	*p*-value	MD (95% CI)	*p*-value	MD (95% CI)	*p*-value	MD (95% CI)	*p*-value	MD (95% CI)	*p*-value	MD (95% CI)	*p*-value
Pre vs. 1st month	−1.73 * (−2.41, −1.05)	<0.001	−0.08 (−0.22, 0.06)	0.831	−1.68 * (−2.20, −1.16)	<0.001	−0.08 (−0.22, 0.06)	0.831	0.00 (-)	-	−0.08 (−0.22, 0.06)	0.831	−0.03 (−0.11, 0.05)	1.000	−0.05 (−0.17, 0.06)	1.000
Pre vs. 2nd month	−3.57 * (−4.92, −2.21)	<0.001	−0.08 (−0.22, 0.06)	0.831	−3.68 * (−4.55, −2.80)	<0.001	−0.08 (−0.22, 0.06)	0.831	0.00 (-)	-	−0.08 (−0.22, 0.06)	0.831	−0.03 (−0.11, 0.05)	1.000	−0.05 (−0.17, 0.06)	1.000
Pre vs. 3rd month	−5.51 * (−7.39, −3.64)	<0.001	−0.41 (−0.99, 0.18)	0.453	−6.08 * (−7.22, −4.94)	<0.001	−0.68 * (−1.30, −0.05)	0.026	−0.11 (−0.30, 0.09)	1.000	−0.19 (−0.47, 0.91)	0.508	−0.24 (−0.62, 0.13)	0.595	−0.14 (−0.37, 0.10)	0.960
Pre vs. post intervention	−5.87 * (−7.83, −3.90)	<0.001	−0.95 (−1.98, 0.90)	0.097	−6.40 * (−7.65, −5.16)	<0.001	−1.24 * (−2.48, −0.01)	0.047	−0.54 (−1.19, 0.11)	0.179	−0.95 * (−1.85, −0.04)	0.035	−0.92 * (−1.71, −0.07)	0.025	−0.76 * (−1.39, −0.13)	0.009
1st month vs. 2nd month	−1.84 * (−2.64, −1.04)	<0.001	0.00 (-)	-	−2.00 * (−2.53, −1.47)	<0.001	0.00 (-)	-	0.00 (-)	-	0.00 (-)	-	0.00 (-)	-	0.00 (-)	-
1st month vs. 3rd month	−3.78 * (−5.11, −2.46)	<0.001	−0.32 (−0.86, 0.21)	0.764	−4.41 * (−5.31, −3.50)	<0.001	−0.60 * (−1.17, −0.02)	0.037	−0.11 (−0.30, 0.09)	1.000	−0.11 (−0.26, 0.05)	0.438	−0.22 (−0.53, 0.09)	0.438	−0.08 (−0.26, 0.10)	1.000
1st month vs. post	−4.14 * (−5.57, −2.71)	<0.001	−0.87 (−1.87, 0.14)	0.143	−4.73 * (−5.70, −3.76)	<0.001	−1.16 (−2.33, 0.01)	0.052	−0.54 (−1.19, 0.11)	0.179	−0.87 (−1.73, 0.01)	0.052	−0.89 * (−1.71, −0.07)	0.024	−0.70 * (−1.31, −0.09)	0.015
2nd month vs. 3rd month	−1.95 * (−2.64, −1.25)	<0.001	−0.32 (−0.86, 0.21)	0.764	−2.41 * (−2.98, −1.83)	<0.001	−0.60 * (−1.17, −0.02)	0.037	−0.11 (−0.30, 0.09)	1.000	−0.11 (−0.26, 0.05)	0.438	−0.22 (−0.53, 0.09)	0.438	−0.08 (−0.26, 0.10)	1.000
2nd month vs. post	−2.30 * (−3.15, −1.45)	<0.001	−0.87 (−1.87, 0.14)	0.143	−2.73 * (−3.31, −2.14)	<0.001	−1.16 (−2.33, 0.01)	0.052	−0.54 (−1.19, 0.11)	0.179	−0.87 (−1.73, 0.01)	0.052	−0.89 * (−1.71, −0.07)	0.024	−0.70 * (−1.31, −0.09)	0.015
3rd month vs. post	−0.35 (−0.76, 0.05)	0.136	−0.54 (−1.21, 0.13)	0.214	−0.32 (−0.71, 0.06)	0.164	−0.57 (−1.38, 0.23)	0.406	−0.43 (−1.06, 0.20)	0.474	−0.76 (−1.61, 0.10)	0.120	−0.68 * (−1.31, −0.04)	0.030	−0.62 * (−1.21, −0.04)	0.030

Repeated measure MANOVA within group was applied, MD = mean difference, 95% CI = 95% confidence interval, * *p*-value < 0.05, vs. = versus.

**Table 5:** Comparisons of mean score of PALMS-M motives within the control group based on time (time effect).

Comparison	Competition		Appearance		Others’ Expectation		Affiliation		Physical Condition		Psychological Condition		Mastery		Enjoyment	
	MD (95% CI)	*p*-value	MD (95% CI)	*p*-value	MD (95% CI)	*p*-value	MD (95% CI)	*p*-value	MD (95% CI)	*p*-value	MD (95% CI)	*p*-value	MD (95% CI)	*p*-value	MD (95% CI)	*p*-value
Pre vs. 1st month	0.18 (−0.06, 0.43)	0.316	0.09 (−0.06, 0.24)	0.831	0.18 (0.02, 0.39)	0.119	0.09 (−0.11, 0.29)	1.000	0.24 (−0.05, 0.54)	0.184	0.21 (−0.74, 0.50)	0.326	0.06 (−0.07, 0.19)	1.000	0.06 (−0.07, 0.19)	1.000
Pre vs. 2nd month	0.24 (−0.08, 0.57)	0.302	0.15 (−0.80, 0.38)	0.575	0.49 * (0.16, 0.81)	0.001	0.09 (−0.11, 0.29)	1.000	0.39 * (0.01, 0.79)	0.048	0.24 (−0.05, 0.54)	0.184	0.06 (−0.07, 0.19)	1.000	0.06 (−0.07, 0.19)	1.000
Pre vs. 3rd month	0.58 * (0.08, 1.07)	0.013	0.27 (−0.06, 0.60)	0.176	0.82 * (0.34, 1.30)	<0.001	0.18 (−0.15, 0.52)	1.000	0.64 * (0.15, 1.12)	0.004	0.46 (−0.02, 0.93)	0.069	0.12 (−0.10, 0.34)	1.000	0.30 (−0.14, 0.75)	0.482
Pre vs. post intervention	1.21 * (0.43, 1.99)	0.001	0.64 (−0.01, 1.28)	0.052	1.27 * (0.76, 1.79)	<0.001	0.36 (−0.22, 0.95)	0.697	1.15 * (0.41, 1.90)	0.001	1.03 * (0.04, 2.02)	0.035	0.42 (−0.28, 1.13)	0.798	0.73 (−0.04, 1.50)	0.075
1st month vs. 2nd month	0.06 (−0.07, 0.19)	1.000	0.06 (−0.07, 0.19)	1.000	0.30 * (0.76, 0.55)	0.007	0.00 (-)	-	0.15 (−0.08, 0.38)	0.575	0.03 (−0.06, 0.12)	1.000	0.00 (-)	-	0.00 (-)	-
1st month vs. 3rd month	0.39 (−0.02, 0.81)	0.072	0.18 (−0.10, 0.46)	0.564	0.64 * (0.76, 1.07)	0.001	0.09 (−0.18, 0.37)	1.000	0.39 * (0.05, 0.74)	0.017	0.24 (−0.11, 0.59)	0.436	0.06 (−0.07, 0.19)	1.000	0.24 (−0.17, 0.66)	0.882
1st month vs. post	1.03 * (−0.28, 1.78)	0.002	0.55 (−0.07, 1.16)	0.119	1.09 * (0.59, 1.59)	<0.001	0.27 (−0.19, 0.73)	0.831	−0.91 * (−1.54, −0.28)	0.001	0.82 (−0.01, 1.64)	0.053	0.36 (−0.25, 0.98)	0.831	0.67 (−0.07, 1.41)	0.104
2nd month vs. 3rd month	0.33 (−0.05, 0.72)	0.139	0.12 (−0.10, 0.34)	1.000	0.33 * (0.20, 0.65)	0.030	0.09 (−0.18, 0.37)	1.000	0.24 (−0.02, 0.51)	0.091	0.21 (−0.07, 0.50)	0.326	0.06 (−0.07, 0.19)	1.000	0.24 (−0.17, 0.66)	0.882
2nd month vs. post	0.97 * (0.23, 1.71)	0.004	0.49 (−0.06, 1.03)	0.112	0.79 * (0.36, 1.22)	<0.001	0.27 (−0.19, 0.73)	0.831	0.76 * (0.19, 1.33)	0.004	0.79 * (0.01, 1.56)	0.043	0.36 (−0.25, 0.98)	0.831	0.67 (−0.07, 1.41)	0.104
3rd month vs. post	0.64 * (0.15, 1.12)	0.004	0.36 (−0.07, 0.80)	0.161	0.46 * (0.16, 0.75)	0.001	0.18 (−0.13, 0.49)	0.184	0.52 * (0.06, 0.97)	0.018	0.58 * (0.02, 1.13)	0.039	0.30 (−0.20, 0.80)	0.766	0.42 * (0.03, 0.82)	0.028

Repeated measure MANOVA within group was applied, MD = mean difference, 95% CI = 95% confidence interval, * *p*-value < 0.05, vs. = versus.

**Table 6:** Comparisons of PALMS-M motives mean score among the two groups based on time (time–group interaction).

Motives	Time	Groups	Mean (95% CI)	Motives	Time	Groups	Mean (95% CI)
Competition	Pre-intervention	Experimental	15.05 (13.74, 16.37)	Physical condition	Pre-intervention	Experimental	19.89 (18.71,21.07)
		Control	17.67 (16.28,19.06)			Control	18.67 (17.42, 19.92)
	1st month	Experimental	16.78 (15.66, 17.91)		1st month	Experimental	19.89 (18.75, 21.04)
		Control	17.49 (16.29, 18.68)			Control	18.42 (17.21, 19.64)
	2nd month	Experimental	18.62 (17.67, 19.58)		2nd month	Experimental	19.89 (18.77, 21.01)
		Control	17.42 (16.41, 18.68)			Control	18.27 (17.08, 19.46)
	3rd month	Experimental	20.57 (19.75, 21.38)		3rd month	Experimental	20.00 (18.93, 21.07)
		Control	17.09 (16.23, 17.95)			Control	18.03 (16.90, 19.17)
	Post-intervention	Experimental	20.92 (20.17, 21.67)		Post-intervention	Experimental	20.43 (19.45, 21.41)
		Control	16.46 (15.66, 17.25)			Control	17.52 (16.48, 18.56)
Appearance	Pre-intervention	Experimental	17.22 (15.71, 18.72)	Psychological condition	Pre-intervention	Experimental	18.05 (16.86, 19.25)
		Control	15.21 (13.62, 16.81)			Control	17.48 (16.22, 18.75)
	1st month	Experimental	17.30 (15.81, 18.78)		1st month	Experimental	18.13 (17.00, 19.27)
		Control	15.12 (13.55, 16.69)			Control	17.27 (16.07, 18.48)
	2nd month	Experimental	17.30 (15.83, 18.76)		2nd month	Experimental	18.14 (17.01, 19.26)
		Control	15.06 (13.51, 16.61)			Control	17.24 (16.05, 18.44)
	3rd month	Experimental	17.62 (16.27, 18.97)		3rd month	Experimental	18.24 (17.17, 19.32)
		Control	14.94 (13.51, 16.37)			Control	17.03 (15.89, 18.17)
	Post-intervention	Experimental	18.16 (17.00, 19.32)		Post-intervention	Experimental	19.00 (18.07, 19.93)
		Control	14.58 (13.35, 15.80)			Control	16.46 (15.47, 17.44)
Others’ expectation	Pre-intervention	Experimental	14.49 (13.61, 15.37)	Mastery	Pre-intervention	Experimental	16.78 (15.45, 18.12)
		Control	16.36 (15.43, 17.30)			Control	15.00 (13.59, 16.41)
	1st month	Experimental	16.16 (15.39, 16.94)		1st month	Experimental	16.81 (15.50, 18.12)
		Control	16.18 (15.36, 17.01)			Control	14.94 (13.55, 16.33)
	2nd month	Experimental	18.16 (17.48, 18.85)		2nd month	Experimental	16.81 (15.50, 18.12)
		Control	15.88 (15.16, 16.60)			Control	14.94 (13.55, 16.33)
	3rd month	Experimental	20.57 (19.94, 21.20)		3rd month	Experimental	17.23 (15.78, 18.27)
		Control	15.55 (14.88, 16.21)			Control	14.88 (13.56, 16.20)
	Post-intervention	Experimental	20.89 (20.30, 21.49)		Post-intervention	Experimental	17.70 (16.59, 18.82)
		Control	15.09 (14.46, 15.72)			Control	14.58 (13.39, 15.76)
Affiliation	Pre-intervention	Experimental	15.43 (14.13, 16.73)	Enjoyment	Pre-intervention	Experimental	17.14 (15.88, 18.40)
		Control	15.09 (13.71, 16.47)			Control	16.64 (15.30, 17.97)
	1st month	Experimental	15.51 (14.25, 16.78)		1st month	Experimental	17.19 (15.94, 18.44)
		Control	15.00 (13.66, 16.34)			Control	16.58 (15.26, 17.90)
	2nd month	Experimental	15.51 (14.25, 16.78)		2nd month	Experimental	17.19 (15.94, 18.44)
		Control	15.00 (13.66, 16.34)			Control	16.58 (15.26, 17.90)
	3rd month	Experimental	16.11 (14.98, 17.24)		3rd month	Experimental	17.27 (16.09, 18.45)
		Control	14.91 (13.71, 16.11)			Control	16.33 (15.09, 17.58)
	Post-intervention	Experimental	16.68 (15.62, 17.73)		Post-intervention	Experimental	17.89 (16.82, 18.97)
		Control	14.73 (13.61, 15.84)			Control	15.91 (14.77, 17.05)

Repeated measure MANOVA between group analysis with regard to time was applied, 95% CI = 95% confidence interval.

**Table 7:** Overall mean difference of amount of physical activity (PA) among experimental and control groups.

Comparison on Amount of PA	Mean Difference (95% CI)	*p*-Value
Experimental-Control	357.77 (148.90, 566.65)	0.001
